# Impact of COVID‐19 on Hematologic Cancer Patients: Insights From the Late Pandemic Phase

**DOI:** 10.1002/cam4.71112

**Published:** 2025-07-31

**Authors:** Daniele Caracciolo, Giuseppe D'Aquino, Caterina Froio, Caterina Romeo, Vincenzo Bosco, Giulia Pensabene, Ludovica Tedesco, Pierosandro Tagliaferri, Pierfrancesco Tassone

**Affiliations:** ^1^ Department of Experimental and Clinical Medicine Magna Græcia University, Campus Salvatore Venuta Catanzaro Italy; ^2^ Medical and Translational Oncology, AOU Renato Dulbecco Catanzaro Italy; ^3^ Department of Medical and Surgical Sciences Magna Graecia University Catanzaro Italy

**Keywords:** chronic lymphocytic leukemia, COVID‐19, hematologic malignancies, late pandemic phase, multiple myeloma, myelodysplastic syndrome, non‐Hodgkin lymphoma

## Abstract

**Background:**

The COVID‐19 pandemic significantly increased mortality risks for individuals with hematological malignancies.

**Patients and Methods:**

We conducted a single‐center observational study, focusing on the late pandemic phase of the infection (2022–2023), to identify risk factors associated with COVID‐19 outcomes in vaccinated patients with hematological malignancies.

**Results:**

Eighty‐five COVID‐19 cases were recorded among patients with hematological malignancies, primarily Multiple Myeloma (MM) (38.8%), chronic lymphocytic leukemia (CLL) (10.6%) and Non‐Hodgkin Lymphoma (NHL) (35.3%). Despite a high COVID‐19 vaccination rate (97.6%), severe/critical illness occurred in 23.5% of patients. The overall COVID‐19‐related mortality rate was 22.4%, with a 30‐day mortality rate of 11.8%. Although mortality rates significantly decreased over the observation time (27.7% vs. 16.7%), this trend was not confirmed in critical infection carriers.

**Conclusion:**

Our data confirm that, despite a reduction in critical infections and overall mortality rates over time, patients with hematological malignancies remain at high risk during the endemic phase of SARS‐CoV‐2 infection.

## 1 Introduction

Five years have passed since the World Health Organization (WHO) declared the onset of the COVID‐19 pandemic. Nevertheless, COVID‐19 continues to pose a significant challenge for patients with hematologic malignancies, a population especially vulnerable to severe viral infections due to their compromised immune systems [1].

In the first phase of the pandemic, before the introduction of effective prophylaxis, many retrospective reports confirmed a more severe clinical outcomes of COVID‐19 in patients with hematological malignancies [2, 3, 4], not only higher than in the non‐cancer population but also higher than in solid tumor carriers [5, 6].

As the pandemic progressed into its late phase, the introduction of vaccines provided a critical tool for mitigating the impact of COVID‐19 [7]. However, the efficacy and safety of these vaccines in patients with hematologic malignancies remain areas of active debate and investigation, warranting focused management [8, 9].

The majority of studies on COVID‐19 in patients with hematologic malignancies have focused on the period between 2020 and 2022 [3, 10]. However, extending the observational timeframe is critically important for several reasons.

First, the continuously evolving landscape of SARS‐CoV‐2 variants necessitates ongoing surveillance to better understand their impact on the clinical course and outcomes in immuno‐compromised patients with hematological malignancies. For instance, a new wave of COVID‐19 was observed in Italy during the second half of 2023, primarily driven by the JN.1 variant, and was associated with an increased number of deaths as compared to the first half of the year [11].

Second, the long‐term outcomes of COVID‐19 in this patient population remain largely undefined. While initial studies provided valuable data on the acute infection and immediate complications, the prevalence and characteristics of long‐term sequelae, including “long COVID,” are not yet fully understood in this vulnerable group [12].

Third, the long‐term effectiveness and safety of COVID‐19 vaccines and antiviral therapies in immunocompromised individuals require further evaluation [13]. Understanding their performance over time is essential to inform treatment and prevention strategies.

Despite these considerations, most available reports on COVID‐19 outcomes in patients with hematologic malignancies do not extend beyond 2022 or fail to cover the entirety of 2023 [14]. This points to a significant research gap regarding the impact of COVID‐19 on this population in the later phases of the pandemic.

In this study, we present a real‐world analysis of the impact of COVID‐19 on hemato‐oncologic patients during the late pandemic phase (2022–2023). By focusing on this specific period, characterized by high vaccination coverage among patients, our findings provide relevant insights into disease management and contribute to improving long‐term outcomes for individuals with hematologic malignancies affected by COVID‐19.

## 2 Patients and Methods

The study was conducted at the Medical Oncology unit of the University Hospital “Renato Dulbecco” of Catanzaro. Clinical data from January 2022 to December 2023, collected from available medical records and described according to methodological quality criteria for case series [11], were processed anonymously according to the Declaration of Helsinki. In accordance with our Institutional Ethical Committee, the study was deemed exempt from ethical approval since only anonymized retrospective data from standard patient care were analyzed. Informed consent for anonymized clinical information for scientific research was provided by all patients at the time of the first visit.

### 2.1 Procedures

To be included in the study, patients had to meet the following inclusion criteria: (a) patient over 18 years of age, (b) active hematological malignancy within the past 5 years before their COVID‐19 diagnosis, (c) laboratory diagnosis for COVID‐19 between January 1, 2022, and December 31, 2023. Data on patients' demographic characteristics and baseline conditions before COVID‐19 were collected. Additional information, such as the reason for the COVID‐19 test, severity of infection, hospitalization after COVID‐19, cause, and day of death were collected.

The diagnosis of COVID‐19 and criteria used to grade infection severity were made according to NIH COVID‐19 Treatment Guidelines (www.covid19treatmentguidelines.nih.gov). Overall mortality was defined as the proportion of any cause deaths as compared to the total number of patients included during the observation time.

### 2.2 Study Objectives

The primary objective of this study was to assess the impact of COVID‐19 on onco‐hematologic patients in terms of epidemiology and clinical outcomes. Secondary objectives were: (1) to evaluate disease severity (i.e., asymptomatic, mild, moderate, severe disease); (2) to estimate the rate of ICU admission; (3) to assess the overall mortality; (4) to stratify patients according to the type of malignancy and type of anti‐cancer therapy.

### 2.3 Statistical Analysis

The primary analysis describes the demographic and clinical characteristics of patients with hematologic malignancy and COVID‐19 diagnosis. Categorical variables are presented with percentages and frequencies, while continuous variables are presented with median, interquartile range (IQR) and absolute range. A univariable Cox regression model was performed to identify variables suspected to independently correlate with the mortality of hematologic malignancy patients with COVID‐19. Variables with a *p*‐value ≤ 0.1 were considered for multivariable analysis by the Cox regression model calculated with the Wald backward method.

Kaplan–Meier survival plots were used for descriptive analysis of mortality. Survival probability of the patients was compared by using the log‐rank test, based on COVID‐19 severity, baseline malignancy, and observation period (semesters of 2022–2023). A *p*‐value ≤ 0.05 was considered statistically significant. SPSSv25.0 was used for analyses (SPSS, IBM Corp., Chicago, IL, United States).

## 3 Results

To address these issues, a total of 90 cases were retrospectively recorded from our institution to identify and evaluate risk factors associated with COVID‐19 outcomes in patients affected by hematologic malignancies during the late pandemic phase (2022–2023). Of these, 5 were excluded for the following reasons: non‐malignant hematological diseases, incomplete information, more than 5 years off therapy from the last chemotherapy. The demographic and clinical characteristics of 85 cases are reported in Table 1. There was a higher prevalence of males (*n* = 48, 56.4%). The median age was 69 years (IQR: 57–75.8; range 22–87). Overall, 31 (42%) patients had at least one comorbidity, with cardiovascular diseases being most frequent (*n* = 56, 69.8%). In 50 patients (67%) smoking history was reported (Table 1).

**TABLE 1:** Baseline characteristics of patients included in the study.

Demographic and clinical characteristics of patients	*n*	%
*Sex*		
Female	37	43.5
Male	48	56.4
*Age*		
Median, years	69	
< 50 years	16	18
> 50 years	69	82
*Comorbidities*		
0–1	56	65.8
> 2	29	34.2
Diabetes mellitus	17	20
Chronic pulmonary disease	7	8.2
Smoking history	50	58.8
Chronic cardiopaty	63	74.1
None	22	25.8
*Malignancy*		
Chronic lymphocytic leukemia	9	10.6
Hodgkin lymphoma	7	8.2
Non‐Hodgkin lymphoma	30	35.3
Multiple myeloma	33	38.8
Chronic myeloproliferative neoplasms	2	2.4
Myelodysplastic syndromes	4	4.7
*Malignancy status before COVID‐19*		
Controlled disease	58	68.2
Active disease	27	31.8
*Severity*		
Asymptomatic	13	15.3
Mild infection	44	51.8
Moderate	8	9.4
Severe infection	16	18.8
Critical infection	4	4.7
*Symptomatology at onset*		
Asymptomatic	13	15.3
Cough	66	77.6
Fever	65	76.4
Dyspnea	20	23.5
Extrapulmonary	14	16.4
*Stay during COVID‐19*		
Hospital	10	11.8
ICU	4	4.7
Home	71	83.5

Patients with Multiple Myeloma (MM) represented the largest subgroup (*n* = 33, 38.8%), followed by patients with non‐Hodgkin lymphoma (NHL) (*n* = 30, 35.3%) and those with Chronic Lymphocytic Leukemia (CLL) (*n* = 9, 10.6%) (Table 1). Active disease was reported in 31.8% of the patients (*n* = 27) (Table 1), and 71.8% of patients (*n* = 61) had received anticancer treatment in the 3 months before the onset of COVID‐19 (Table S2). The most frequent treatments were target therapy administered to 35 patients (41.2%), followed by chemotherapy with immunotherapy (*n* = 30, 35.3%) or chemotherapy alone administered to 8 patients (9.4%). Four patients (4.7%) had a transplant procedure performed in their clinical history (all autologous HSCT) (Table S1).

SARS‐CoV‐2 infection was diagnosed by nasopharyngeal swab in all patients. COVID‐19 tests were performed in 75 patients (89%) because of pulmonary and/or extra‐pulmonary symptoms, and in 10 patients (11%) as part of asymptomatic screening. The presence of respiratory symptoms, mainly cough and fever, was the most frequent clinical presentation, reported in 66 (76.3%) and 65 (74.7%) respectively, and in 12 of them (15%) it occurred together with extra‐pulmonary symptoms (Table 1). The rate of COVID‐19 vaccination was 97.6% (83/85). The majority of patients received an mRNA vaccine (BioNTech/Pfizer *n* = 78 [94%], Moderna *n* = 3 [3.6%]), whereas the remaining 2 (2.4%) received a vector‐based vaccine (AstraZeneca Oxford); overall, the median time from the last dose of the vaccine to COVID‐19 diagnosis was 150 days (IQR: 117–210). Seventy‐nine (95.2%) patients received more than 2 doses, whereas the remaining 4 (4.8%) received only 1 shot.

COVID‐19 infection was determined to be critical in 4 patients (4.7%), severe in 16 (18.8%), moderate in 8 (9.4%), mild in 44 (51.8%), asymptomatic in 13 (15.3%) (Table 1). Overall, 10 patients (11.8%) were hospitalized and 4 (4.7%) required hospitalization in an ICU (Table 1).

**FIGURE 1:**
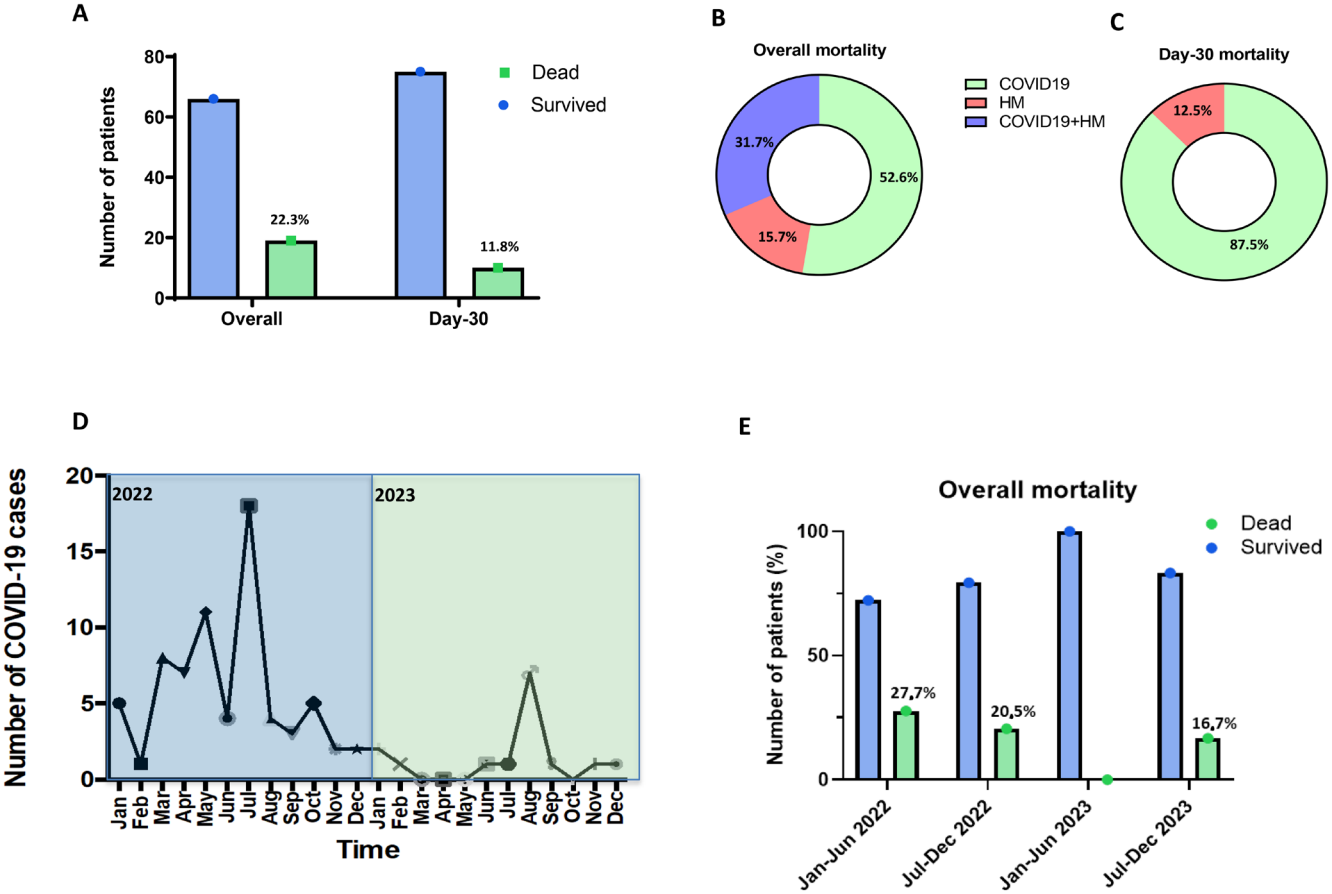
COVID‐19 mortality in hematologic malignancies (HM). (A) Overall and 30 day after‐infection mortality. (B, C) Overall (B) and Day‐30 (C) mortality cause of death. (D, E) Number of COVID‐19 cases (D) and overall mortality (E) during 2022 and 2023 semesters.

Altogether, during the observation phase, 19 patients (22.3%) died, while the mortality at 30 days from infection (Day‐30 mortality) rate was 11.8% (*n* = 10) (Figure 1A and Table S2). The primary cause of overall mortality was COVID‐19 in 10 patients (52.6%), hematological malignancy in 3 patients (15.7%), and the combination of COVID‐19 and progressive hematological malignancy in 6 patients (31.7%) (Figure 1B), while COVID‐19 was associated with Day‐30 mortality in 87.5% of cases (Figure 1C). Patients over the age of 70 years had the highest mortality (38.1%) as compared to younger patients (51–69 years, 10.7%) (*p* < 0.01). Considering the different hematologic malignancies, the higher number of fatalities was observed in myelodysplastic syndromes (MDS) 2/4 (50%), followed by MM 9/33 (27.3%) and NHL 6/30 (20%) (*p* < 0.05) (Table S2).

Regarding last underlying treatments for hematologic malignancy before COVID‐19, the highest mortality rate was observed among patients receiving demethylating agents (2/4, 50%), followed by target therapy (10/35, 28.5%) and chemo‐immunotherapy (6/30, 20%) (*p* < 0.05) (Table S2).

Patients with critical COVID‐19 died in a higher proportion (4/4, 100%) than those with severe [(31.3%, 5/11), *p* < 0.01] or mild infection [(7/37, 7%), *p* < 0.01]. A higher mortality rate was reported for patients admitted to ICU (3/4, 75%), compared to non‐ICU patients [(16/81, 19.7%) *p* < 0.001] and for patients with active hematologic malignancy (17/27, 63%) at the time of COVID‐19 diagnosis as compared to those with stable disease [(2/58, 3.4%), *p* < 0.01] (Figure S1A–C and Table S2).

During the study period from 2022 to 2023, the peak of infection diagnosis was registered in July 2022 (19/85, 22%) (Figure 1D). A decrease in the overall mortality from 2022 to 2023 was observed (Figure 1E), even when considering the type of baseline malignancy and anti‐cancer treatment. However, this reduction was more limited for ICU‐admitted and critical patients (Tables S3 and S5).

In the univariable COX regression analysis, multiple factors negatively influenced mortality risk (Table S4A). In the multivariable analysis, the following parameters were significantly associated with higher mortality: age increase (> 70 years), active disease, and ICU admission (Table S4B).

## 4 Discussion

Overall, this monocentric real‐world study describes the demographic and clinical characteristics of patients with hematologic malignancies who developed COVID‐19, with a special focus on the late pandemic phase of infection (2022–2023), in which the majority of subjects received anti‐COVID19 specific vaccine and new therapeutic strategies are available [15]. Multiple Myeloma and non‐Hodgkin lymphoma were the most common types of hematologic malignancies among the population included, and more than 70% of the patients had received anti‐neoplastic therapy in the 3 months before the onset of COVID‐19.

The most frequent treatments administered to the patients were target therapy followed by chemo‐immunotherapy. The peak of COVID‐19 infection among the patients was observed in July 2022, which could be attributed to the timing of the administration of the last dose of the vaccine in most patients (December 2021, *data not shown*). The most common clinical presentation of COVID‐19 among the patients was respiratory symptoms, mainly cough and fever.

The overall mortality rate was 22.3%, while Day‐30 mortality was 11.8% with COVID‐19 being associated with mortality in 87.5% of these cases.

Our analysis identified several factors that negatively influenced mortality, such as increasing age, active disease, and ICU admission.

The study also revealed a mild decline in the severity and mortality of COVID‐19 cases as time progressed. However, despite positive trends and progress, the mortality of COVID‐19 persists for hematological malignancy patients, particularly those who are critically ill, where the mortality rate ranges from 75% to 100%. In addition, it is important to note that COVID‐19 diagnosis is often delayed active treatments for hematologic malignancies, further impacting the overall mortality rate of this population. Indeed, our analysis shows that in 47.4% of cases, progressing hematologic malignancy strongly influenced COVID‐19 mortality.

Our results are in line with recent reports describing outcomes of COVID‐19 infection in onco‐hematological SARS‐COV2‐vaccinated patients. In particular, the European Hematology Association's Specialized Working Group, Infections in Hematology (EPICOVIDEHA Registry) [10], reported a 30‐day mortality of 9.2%, which was significantly lower compared to the pre‐vaccine period (approximately 31%). Furthermore, the INFORM study highlighted that immunocompromised patients continued to be disproportionately affected by COVID19 with 22% of hospitalizations, 28% of ICU admissions, and 24% of overall mortality, despite a vaccination rate of 84% [16].

However, all of these reports limit their observation period to 2022, revealing a clear research gap in the literature regarding the evolution of pandemic outcomes in patients with hematologic malignancies beyond this timeframe [17].

Indeed, although WHO has declared the end of the pandemic phase of COVID‐19 on May 5 2023 [18], significant concerns remain regarding the safe return to normal life, particularly in frail/vulnerable populations such as patients with hematologic malignancies and those with the risk factors highlighted in our report. Notably, a new wave of COVID‐19 affected Italy in the second half of 2023, leading to an increase in the mortality rate.

In this context, our study shed light on the impact of COVID‐19 infection in patients with hematologic malignancies during the very late phase of the pandemic (2022–2023), confirming that older age, critical infection, and active disease were significantly associated with increased mortality.

The higher number of fatalities observed in myelodysplastic syndromes (MDS), although limited from a small number of cases included, is in line with larger studies [2, 19, 20].

Our study has several limitations: the use of retrospective data from a single institution, small sample size, limited number of certain subgroups (e.g., ICU, MDS), short observation time, lack of some laboratory monitoring data, and viral genome analysis. Furthermore, it is likely that for 2023, our observations did not include patients with mild infections who may not have sought medical attention and therefore not performed tests for diagnosis.

## 5 Conclusion

Despite its limitations, our study provides valuable real‐world insights into COVID‐19 outcomes among patients with hematologic malignancies and identifies independent risk factors in the late phase of the pandemic. To our knowledge, this is the first report that extends observation into 2023. As the COVID‐19 landscape continues to evolve and new variants emerge, sustained effort in research, clinical adaptation, and patient advocacy will be essential to improving outcomes for this high‐risk population.

## Author Contributions

**Daniele Caracciolo:** conceptualization, investigation, methodology, formal analysis, writing – original draft. **Giuseppe D'Aquino:** investigation, data curation. **Caterina Froio:** investigation, data curation. **Caterina Romeo:** investigation, data curation. **Vincenzo Bosco:** writing – review and editing. **Giulia Pensabene:** writing – review and editing. **Ludovica Tedesco:** writing – review and editing. **Pierosandro Tagliaferri:** conceptualization, writing – original draft, supervision. **Pierfrancesco Tassone:** conceptualization, writing – original draft, supervision.

## Ethics Statement

The study was deemed exempt from ethical approval since only anonymized retrospective data from standard patient care were analyzed, in accordance with our Institutional Ethical Committee.

## Consent

Informed consent for anonymized clinical information for scientific research was provided by all patients at the time of first visit.

## Conflicts of Interest

The authors declare no conflicts of interest.

## Supporting information


**Figure S1:** Survival probability per status of malignancy at COVID‐19 diagnosis (A), hospitalization (B), and COVID‐19 severity (C).

**Table S1:** Summary of last received hematologic treatment at COVID‐19 diagnosis.

**Table S2:** Overall mortality by baseline characteristics and treatment received.

**Table S3:** Overall mortality and hospitalization rate from 2022 to 2023.

**Table S4:** Factors associated to COVID‐19 mortality.

**Table S5:** COVID‐19 impact from 2022 to 2023.

## Data Availability

Data sharing not applicable to this article as no datasets were generated or analysed during the current study.
