# Supplementary material for: Impact of COVID‐19 on Hematologic Cancer Patients: Insights From the Late Pandemic Phase
Source: Cancer Med. 2025 Jul 31;14(15):e71112. doi: 10.1002/cam4.71112 (PMC12311482; doi:10.1002/cam4.71112)
Supplement: Supplementary file 1 — Figure S1: Survival probability per status of malignancy at COVID‐19 diagnosis (A), hospitalization (B), and COVID‐19 severity (C). [file CAM4-14-e71112-s003.pptx]

## Slide 1
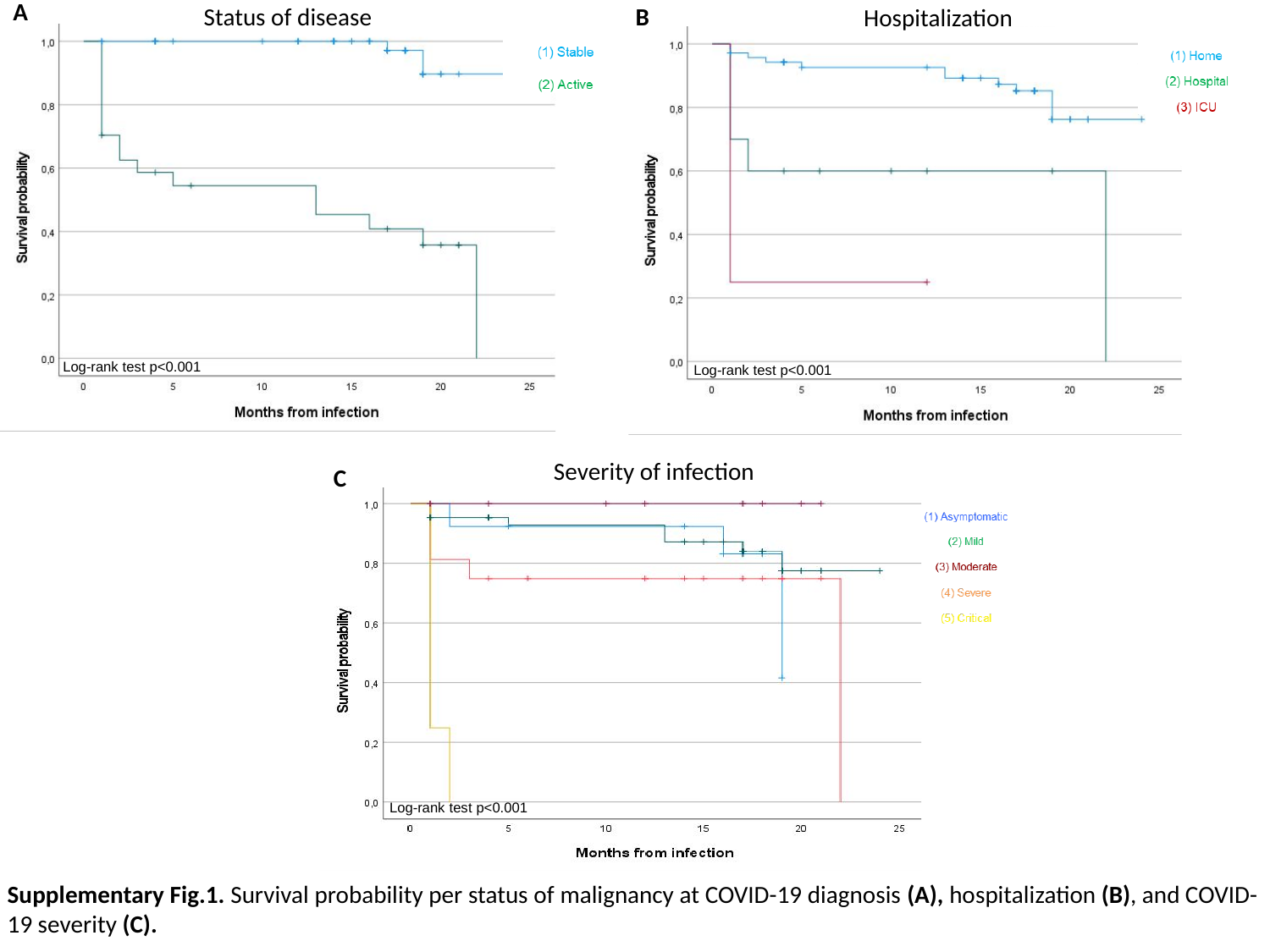

A
 B
Status of disease
Hospitalization
Log-rank test p<0.001
Log-rank test p<0.001
Severity of infection
 C
Log-rank test p<0.001
Supplementary Fig.1. Survival probability per status of malignancy at COVID-19 diagnosis (A), hospitalization (B), and COVID-19 severity (C).
