# Supplementary material for: Impact of COVID‐19 on Hematologic Cancer Patients: Insights From the Late Pandemic Phase
Source: Cancer Med. 2025 Jul 31;14(15):e71112. doi: 10.1002/cam4.71112 (PMC12311482; doi:10.1002/cam4.71112)
Supplement: Supplementary file 2 — Table S1: Summary of last received hematologic treatment at COVID‐19 diagnosis. [file CAM4-14-e71112-s002.pptx]

## Slide 1
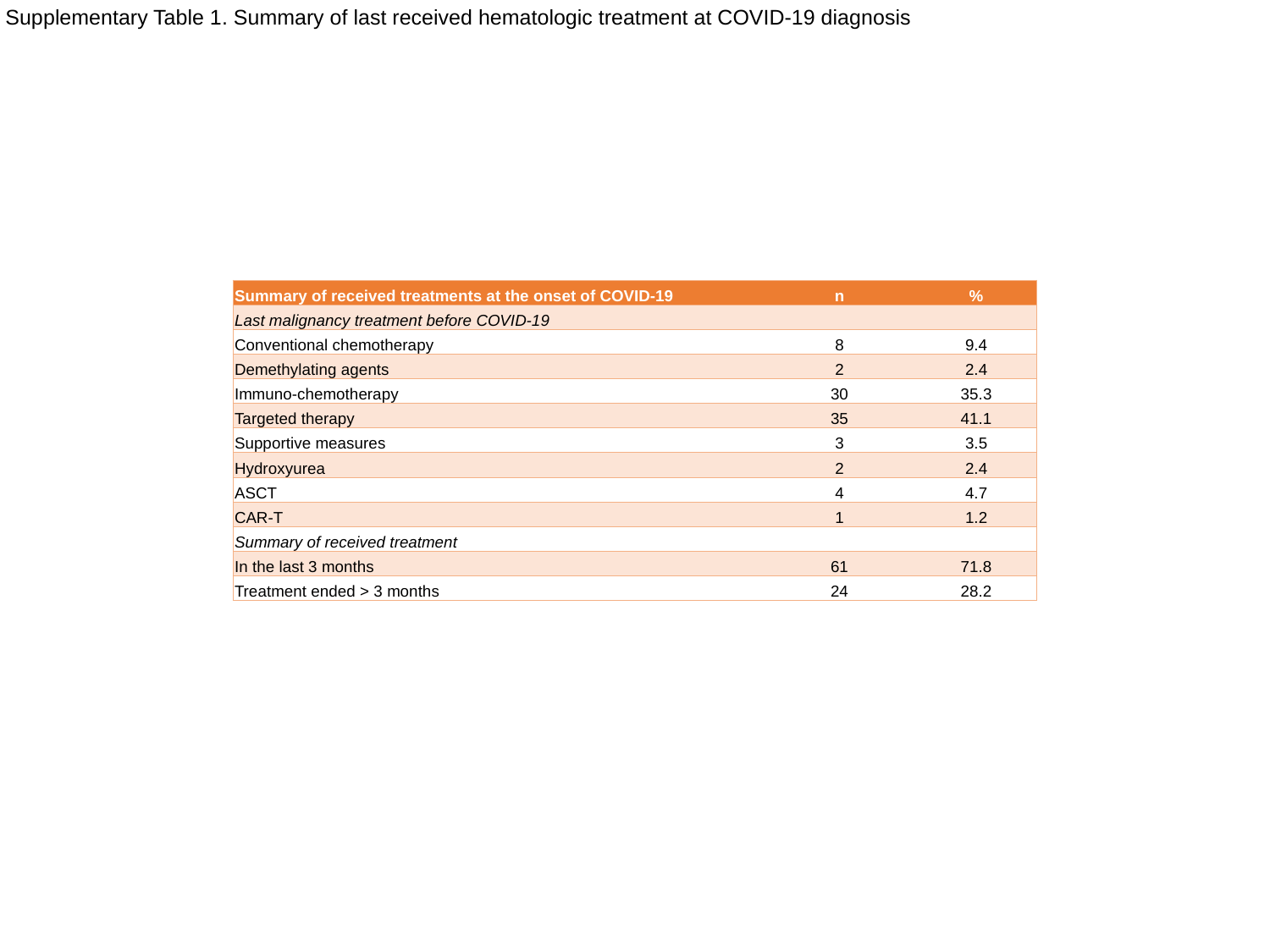

Supplementary Table 1. Summary of last received hematologic treatment at COVID-19 diagnosis
| Summary of received treatments at the onset of COVID-19 | n | % |
| --- | --- | --- |
| Last malignancy treatment before COVID-19 | | |
| Conventional chemotherapy | 8 | 9.4 |
| Demethylating agents | 2 | 2.4 |
| Immuno-chemotherapy | 30 | 35.3 |
| Targeted therapy | 35 | 41.1 |
| Supportive measures | 3 | 3.5 |
| Hydroxyurea | 2 | 2.4 |
| ASCT | 4 | 4.7 |
| CAR-T | 1 | 1.2 |
| Summary of received treatment | | |
| In the last 3 months | 61 | 71.8 |
| Treatment ended > 3 months | 24 | 28.2 |
