# Supplementary material for: Impact of COVID‐19 on Hematologic Cancer Patients: Insights From the Late Pandemic Phase
Source: Cancer Med. 2025 Jul 31;14(15):e71112. doi: 10.1002/cam4.71112 (PMC12311482; doi:10.1002/cam4.71112)
Supplement: Supplementary file 3 — Table S2: Overall mortality by baseline characteristics and treatment received. [file CAM4-14-e71112-s001.pptx]

## Slide 1
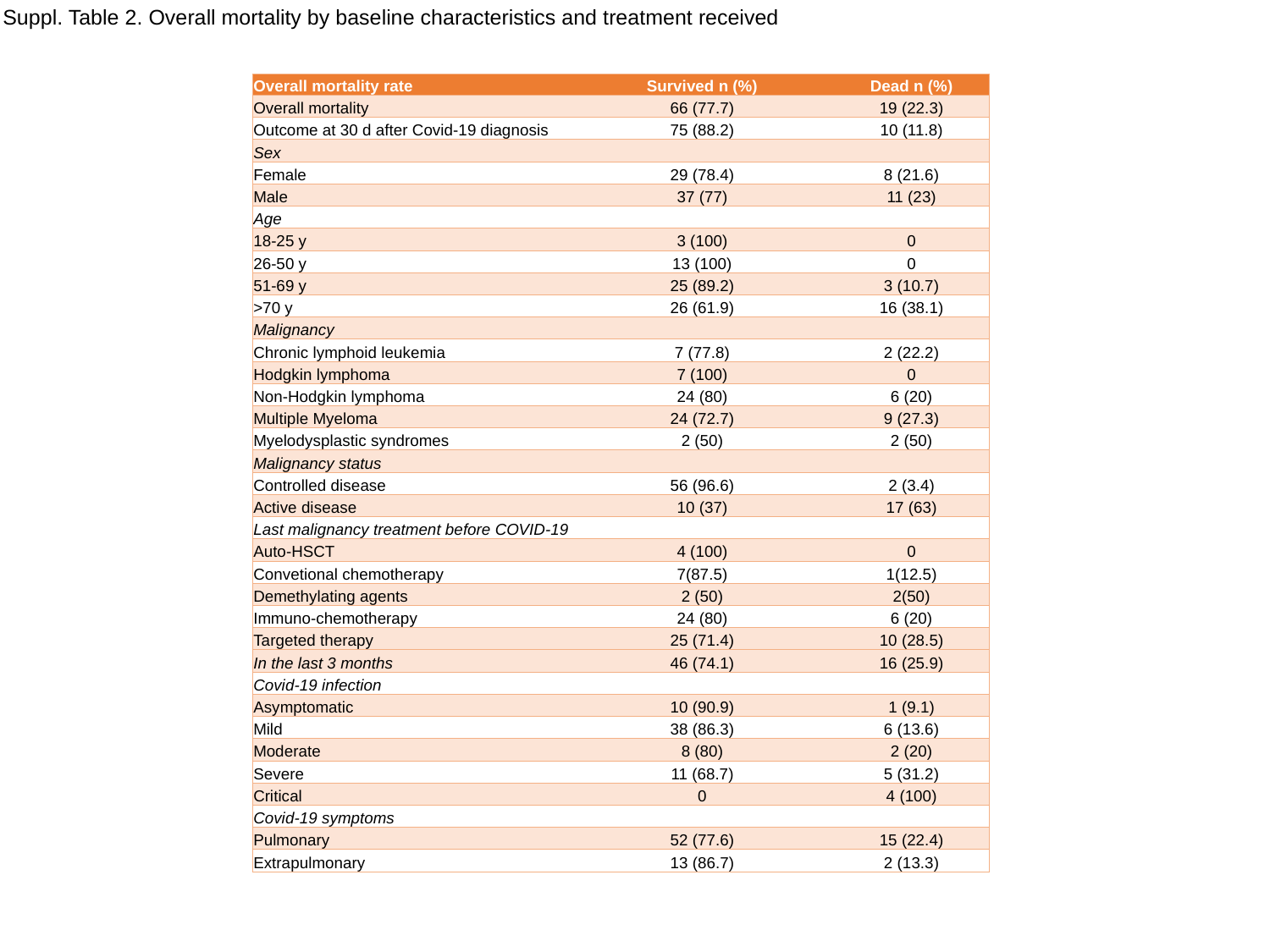

Suppl. Table 2. Overall mortality by baseline characteristics and treatment received
| Overall mortality rate | Survived n (%) | Dead n (%) |
| --- | --- | --- |
| Overall mortality | 66 (77.7) | 19 (22.3) |
| Outcome at 30 d after Covid-19 diagnosis | 75 (88.2) | 10 (11.8) |
| Sex | | |
| Female | 29 (78.4) | 8 (21.6) |
| Male | 37 (77) | 11 (23) |
| Age | | |
| 18-25 y | 3 (100) | 0 |
| 26-50 y | 13 (100) | 0 |
| 51-69 y | 25 (89.2) | 3 (10.7) |
| >70 y | 26 (61.9) | 16 (38.1) |
| Malignancy | | |
| Chronic lymphoid leukemia | 7 (77.8) | 2 (22.2) |
| Hodgkin lymphoma | 7 (100) | 0 |
| Non-Hodgkin lymphoma | 24 (80) | 6 (20) |
| Multiple Myeloma | 24 (72.7) | 9 (27.3) |
| Myelodysplastic syndromes | 2 (50) | 2 (50) |
| Malignancy status | | |
| Controlled disease | 56 (96.6) | 2 (3.4) |
| Active disease | 10 (37) | 17 (63) |
| Last malignancy treatment before COVID-19 | | |
| Auto-HSCT | 4 (100) | 0 |
| Convetional chemotherapy | 7(87.5) | 1(12.5) |
| Demethylating agents | 2 (50) | 2(50) |
| Immuno-chemotherapy | 24 (80) | 6 (20) |
| Targeted therapy | 25 (71.4) | 10 (28.5) |
| In the last 3 months | 46 (74.1) | 16 (25.9) |
| Covid-19 infection | | |
| Asymptomatic | 10 (90.9) | 1 (9.1) |
| Mild | 38 (86.3) | 6 (13.6) |
| Moderate | 8 (80) | 2 (20) |
| Severe | 11 (68.7) | 5 (31.2) |
| Critical | 0 | 4 (100) |
| Covid-19 symptoms | | |
| Pulmonary | 52 (77.6) | 15 (22.4) |
| Extrapulmonary | 13 (86.7) | 2 (13.3) |
