# Supplementary material for: Impact of COVID‐19 on Hematologic Cancer Patients: Insights From the Late Pandemic Phase
Source: Cancer Med. 2025 Jul 31;14(15):e71112. doi: 10.1002/cam4.71112 (PMC12311482; doi:10.1002/cam4.71112)
Supplement: Supplementary file 4 — Table S3: Overall mortality and hospitalization rate from 2022 to 2023. [file CAM4-14-e71112-s004.pptx]

## Slide 1
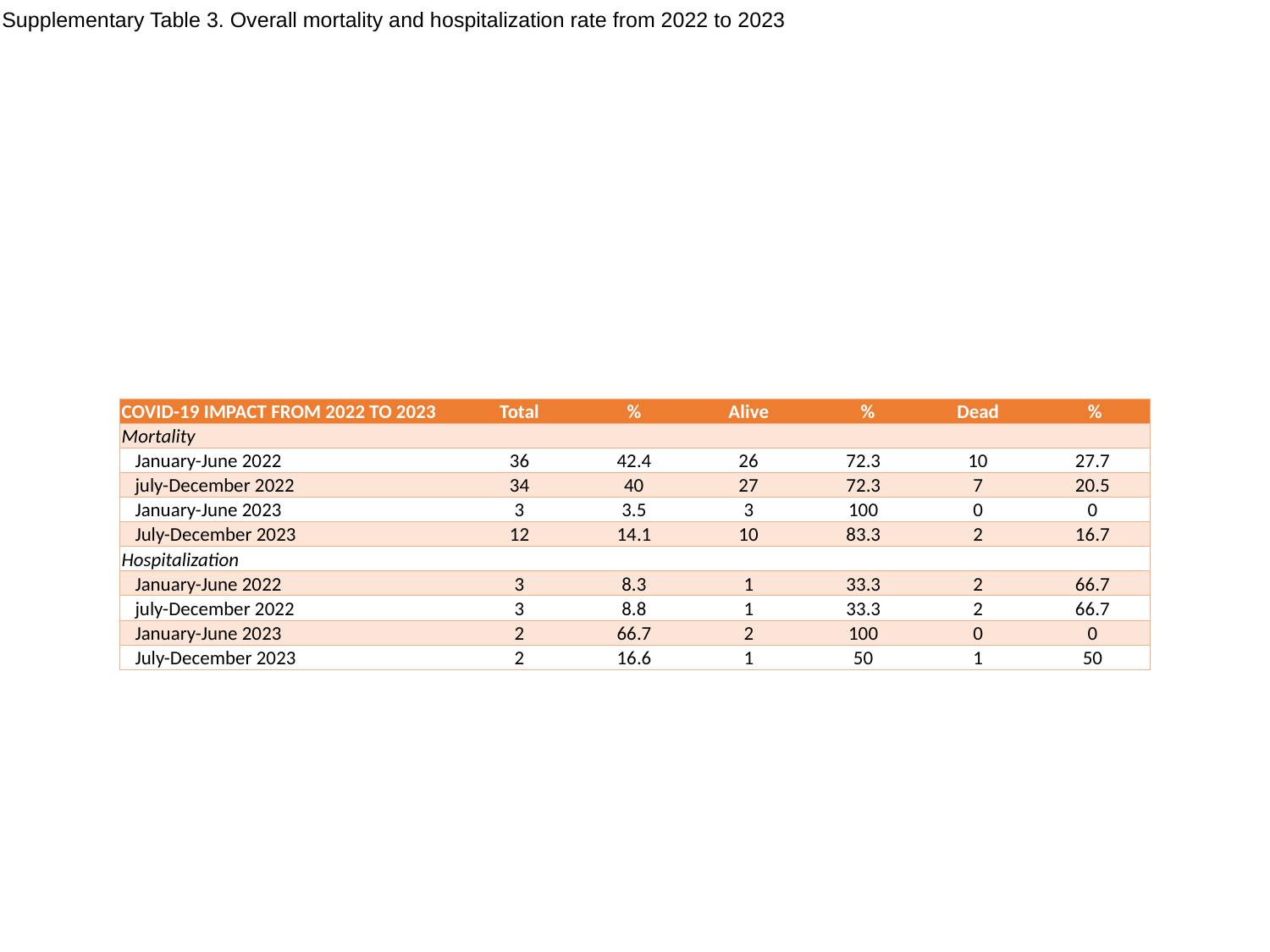

Supplementary Table 3. Overall mortality and hospitalization rate from 2022 to 2023
| COVID-19 IMPACT FROM 2022 TO 2023 | Total | % | Alive | % | Dead | % |
| --- | --- | --- | --- | --- | --- | --- |
| Mortality | | | | | | |
| January-June 2022 | 36 | 42.4 | 26 | 72.3 | 10 | 27.7 |
| july-December 2022 | 34 | 40 | 27 | 72.3 | 7 | 20.5 |
| January-June 2023 | 3 | 3.5 | 3 | 100 | 0 | 0 |
| July-December 2023 | 12 | 14.1 | 10 | 83.3 | 2 | 16.7 |
| Hospitalization | | | | | | |
| January-June 2022 | 3 | 8.3 | 1 | 33.3 | 2 | 66.7 |
| july-December 2022 | 3 | 8.8 | 1 | 33.3 | 2 | 66.7 |
| January-June 2023 | 2 | 66.7 | 2 | 100 | 0 | 0 |
| July-December 2023 | 2 | 16.6 | 1 | 50 | 1 | 50 |
