# Supplementary material for: Impact of COVID‐19 on Hematologic Cancer Patients: Insights From the Late Pandemic Phase
Source: Cancer Med. 2025 Jul 31;14(15):e71112. doi: 10.1002/cam4.71112 (PMC12311482; doi:10.1002/cam4.71112)
Supplement: Supplementary file 5 — Table S4: Factors associated to COVID‐19 mortality. [file CAM4-14-e71112-s006.pptx]

## Slide 1
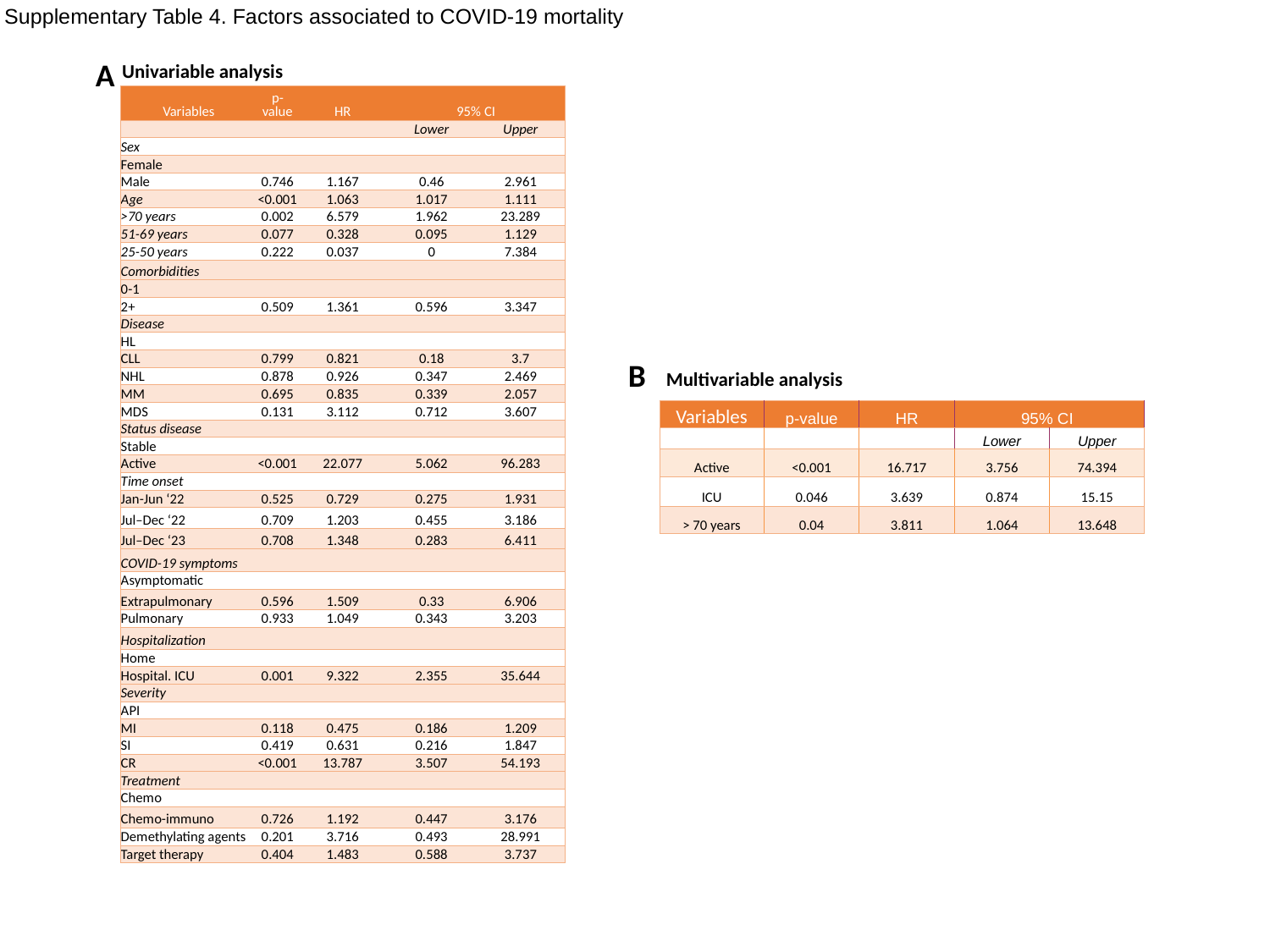

Supplementary Table 4. Factors associated to COVID-19 mortality
A
Univariable analysis
| Variables | p-value | HR | 95% CI | |
| --- | --- | --- | --- | --- |
| | | | Lower | Upper |
| Sex | | | | |
| Female | | | | |
| Male | 0.746 | 1.167 | 0.46 | 2.961 |
| Age | <0.001 | 1.063 | 1.017 | 1.111 |
| >70 years | 0.002 | 6.579 | 1.962 | 23.289 |
| 51-69 years | 0.077 | 0.328 | 0.095 | 1.129 |
| 25-50 years | 0.222 | 0.037 | 0 | 7.384 |
| Comorbidities | | | | |
| 0-1 | | | | |
| 2+ | 0.509 | 1.361 | 0.596 | 3.347 |
| Disease | | | | |
| HL | | | | |
| CLL | 0.799 | 0.821 | 0.18 | 3.7 |
| NHL | 0.878 | 0.926 | 0.347 | 2.469 |
| MM | 0.695 | 0.835 | 0.339 | 2.057 |
| MDS | 0.131 | 3.112 | 0.712 | 3.607 |
| Status disease | | | | |
| Stable | | | | |
| Active | <0.001 | 22.077 | 5.062 | 96.283 |
| Time onset | | | | |
| Jan-Jun ‘22 | 0.525 | 0.729 | 0.275 | 1.931 |
| Jul–Dec ‘22 | 0.709 | 1.203 | 0.455 | 3.186 |
| Jul–Dec ‘23 | 0.708 | 1.348 | 0.283 | 6.411 |
| COVID-19 symptoms | | | | |
| Asymptomatic | | | | |
| Extrapulmonary | 0.596 | 1.509 | 0.33 | 6.906 |
| Pulmonary | 0.933 | 1.049 | 0.343 | 3.203 |
| Hospitalization | | | | |
| Home | | | | |
| Hospital. ICU | 0.001 | 9.322 | 2.355 | 35.644 |
| Severity | | | | |
| API | | | | |
| MI | 0.118 | 0.475 | 0.186 | 1.209 |
| SI | 0.419 | 0.631 | 0.216 | 1.847 |
| CR | <0.001 | 13.787 | 3.507 | 54.193 |
| Treatment | | | | |
| Chemo | | | | |
| Chemo-immuno | 0.726 | 1.192 | 0.447 | 3.176 |
| Demethylating agents | 0.201 | 3.716 | 0.493 | 28.991 |
| Target therapy | 0.404 | 1.483 | 0.588 | 3.737 |
B
Multivariable analysis
| Variables | p-value | HR | 95% CI | |
| --- | --- | --- | --- | --- |
| | | | Lower | Upper |
| Active | <0.001 | 16.717 | 3.756 | 74.394 |
| ICU | 0.046 | 3.639 | 0.874 | 15.15 |
| > 70 years | 0.04 | 3.811 | 1.064 | 13.648 |
