# Supplementary material for: Impact of COVID‐19 on Hematologic Cancer Patients: Insights From the Late Pandemic Phase
Source: Cancer Med. 2025 Jul 31;14(15):e71112. doi: 10.1002/cam4.71112 (PMC12311482; doi:10.1002/cam4.71112)
Supplement: Supplementary file 6 — Table S5: COVID‐19 impact from 2022 to 2023. [file CAM4-14-e71112-s005.pptx]

## Slide 1
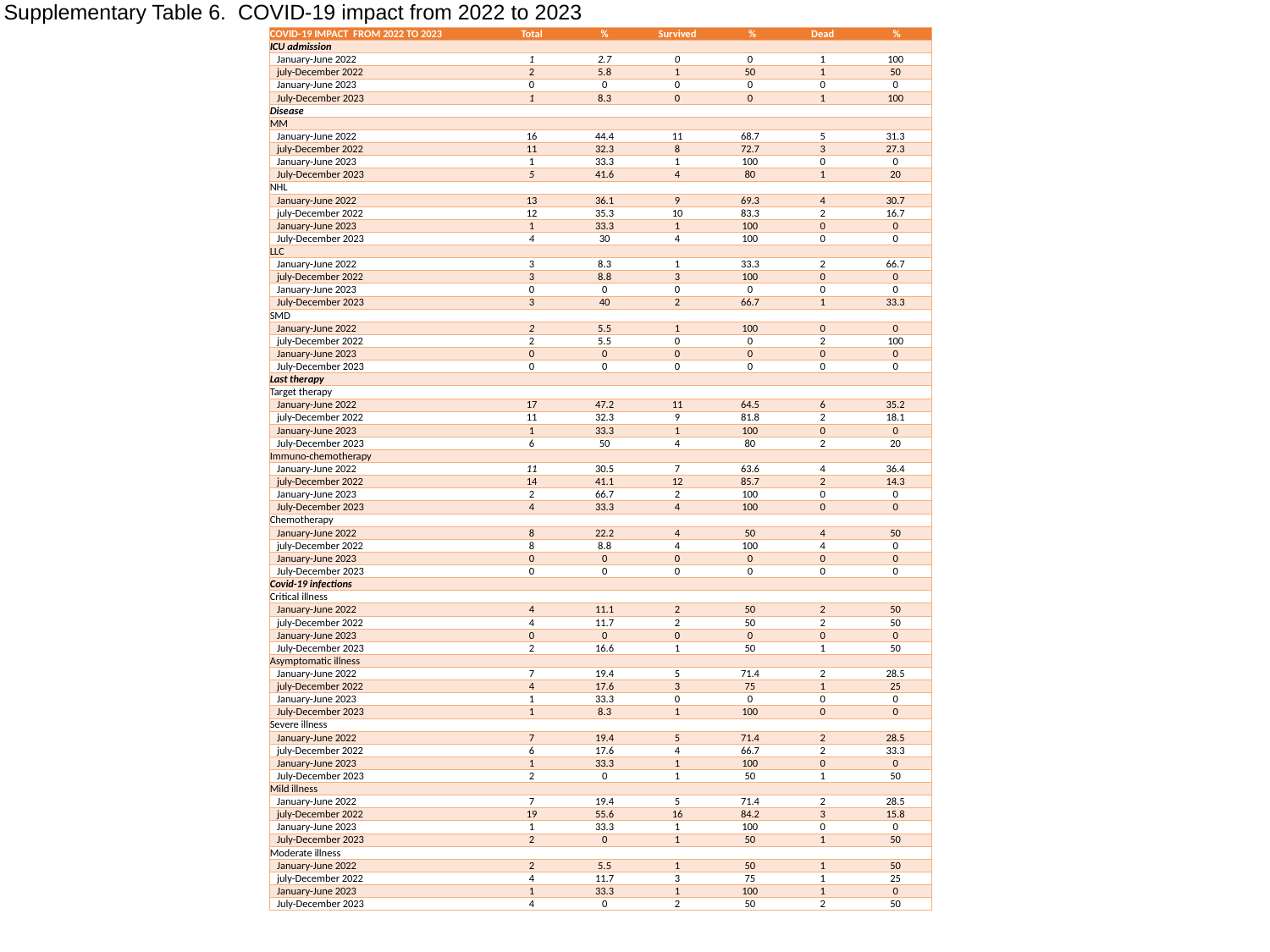

Supplementary Table 6. COVID-19 impact from 2022 to 2023
| COVID-19 IMPACT FROM 2022 TO 2023 | Total | % | Survived | % | Dead | % |
| --- | --- | --- | --- | --- | --- | --- |
| ICU admission | | | | | | |
| January-June 2022 | 1 | 2.7 | 0 | 0 | 1 | 100 |
| july-December 2022 | 2 | 5.8 | 1 | 50 | 1 | 50 |
| January-June 2023 | 0 | 0 | 0 | 0 | 0 | 0 |
| July-December 2023 | 1 | 8.3 | 0 | 0 | 1 | 100 |
| Disease | | | | | | |
| MM | | | | | | |
| January-June 2022 | 16 | 44.4 | 11 | 68.7 | 5 | 31.3 |
| july-December 2022 | 11 | 32.3 | 8 | 72.7 | 3 | 27.3 |
| January-June 2023 | 1 | 33.3 | 1 | 100 | 0 | 0 |
| July-December 2023 | 5 | 41.6 | 4 | 80 | 1 | 20 |
| NHL | | | | | | |
| January-June 2022 | 13 | 36.1 | 9 | 69.3 | 4 | 30.7 |
| july-December 2022 | 12 | 35.3 | 10 | 83.3 | 2 | 16.7 |
| January-June 2023 | 1 | 33.3 | 1 | 100 | 0 | 0 |
| July-December 2023 | 4 | 30 | 4 | 100 | 0 | 0 |
| LLC | | | | | | |
| January-June 2022 | 3 | 8.3 | 1 | 33.3 | 2 | 66.7 |
| july-December 2022 | 3 | 8.8 | 3 | 100 | 0 | 0 |
| January-June 2023 | 0 | 0 | 0 | 0 | 0 | 0 |
| July-December 2023 | 3 | 40 | 2 | 66.7 | 1 | 33.3 |
| SMD | | | | | | |
| January-June 2022 | 2 | 5.5 | 1 | 100 | 0 | 0 |
| july-December 2022 | 2 | 5.5 | 0 | 0 | 2 | 100 |
| January-June 2023 | 0 | 0 | 0 | 0 | 0 | 0 |
| July-December 2023 | 0 | 0 | 0 | 0 | 0 | 0 |
| Last therapy | | | | | | |
| Target therapy | | | | | | |
| January-June 2022 | 17 | 47.2 | 11 | 64.5 | 6 | 35.2 |
| july-December 2022 | 11 | 32.3 | 9 | 81.8 | 2 | 18.1 |
| January-June 2023 | 1 | 33.3 | 1 | 100 | 0 | 0 |
| July-December 2023 | 6 | 50 | 4 | 80 | 2 | 20 |
| Immuno-chemotherapy | | | | | | |
| January-June 2022 | 11 | 30.5 | 7 | 63.6 | 4 | 36.4 |
| july-December 2022 | 14 | 41.1 | 12 | 85.7 | 2 | 14.3 |
| January-June 2023 | 2 | 66.7 | 2 | 100 | 0 | 0 |
| July-December 2023 | 4 | 33.3 | 4 | 100 | 0 | 0 |
| Chemotherapy | | | | | | |
| January-June 2022 | 8 | 22.2 | 4 | 50 | 4 | 50 |
| july-December 2022 | 8 | 8.8 | 4 | 100 | 4 | 0 |
| January-June 2023 | 0 | 0 | 0 | 0 | 0 | 0 |
| July-December 2023 | 0 | 0 | 0 | 0 | 0 | 0 |
| Covid-19 infections | | | | | | |
| Critical illness | | | | | | |
| January-June 2022 | 4 | 11.1 | 2 | 50 | 2 | 50 |
| july-December 2022 | 4 | 11.7 | 2 | 50 | 2 | 50 |
| January-June 2023 | 0 | 0 | 0 | 0 | 0 | 0 |
| July-December 2023 | 2 | 16.6 | 1 | 50 | 1 | 50 |
| Asymptomatic illness | | | | | | |
| January-June 2022 | 7 | 19.4 | 5 | 71.4 | 2 | 28.5 |
| july-December 2022 | 4 | 17.6 | 3 | 75 | 1 | 25 |
| January-June 2023 | 1 | 33.3 | 0 | 0 | 0 | 0 |
| July-December 2023 | 1 | 8.3 | 1 | 100 | 0 | 0 |
| Severe illness | | | | | | |
| January-June 2022 | 7 | 19.4 | 5 | 71.4 | 2 | 28.5 |
| july-December 2022 | 6 | 17.6 | 4 | 66.7 | 2 | 33.3 |
| January-June 2023 | 1 | 33.3 | 1 | 100 | 0 | 0 |
| July-December 2023 | 2 | 0 | 1 | 50 | 1 | 50 |
| Mild illness | | | | | | |
| January-June 2022 | 7 | 19.4 | 5 | 71.4 | 2 | 28.5 |
| july-December 2022 | 19 | 55.6 | 16 | 84.2 | 3 | 15.8 |
| January-June 2023 | 1 | 33.3 | 1 | 100 | 0 | 0 |
| July-December 2023 | 2 | 0 | 1 | 50 | 1 | 50 |
| Moderate illness | | | | | | |
| January-June 2022 | 2 | 5.5 | 1 | 50 | 1 | 50 |
| july-December 2022 | 4 | 11.7 | 3 | 75 | 1 | 25 |
| January-June 2023 | 1 | 33.3 | 1 | 100 | 1 | 0 |
| July-December 2023 | 4 | 0 | 2 | 50 | 2 | 50 |
